# Supplementary material for: Alternative CUG Codon Usage in the Halotolerant Yeast Debaryomyces hansenii: Gene Expression Profiles Provide New Insights into Ambiguous Translation
Source: J Fungi (Basel). 2022 Sep 16;8(9):970. doi: 10.3390/jof8090970 (PMC9502446; doi:10.3390/jof8090970)
Supplement: Supplementary file 1 [file jof-08-00970-s001.zip › Figure S1.pdf]

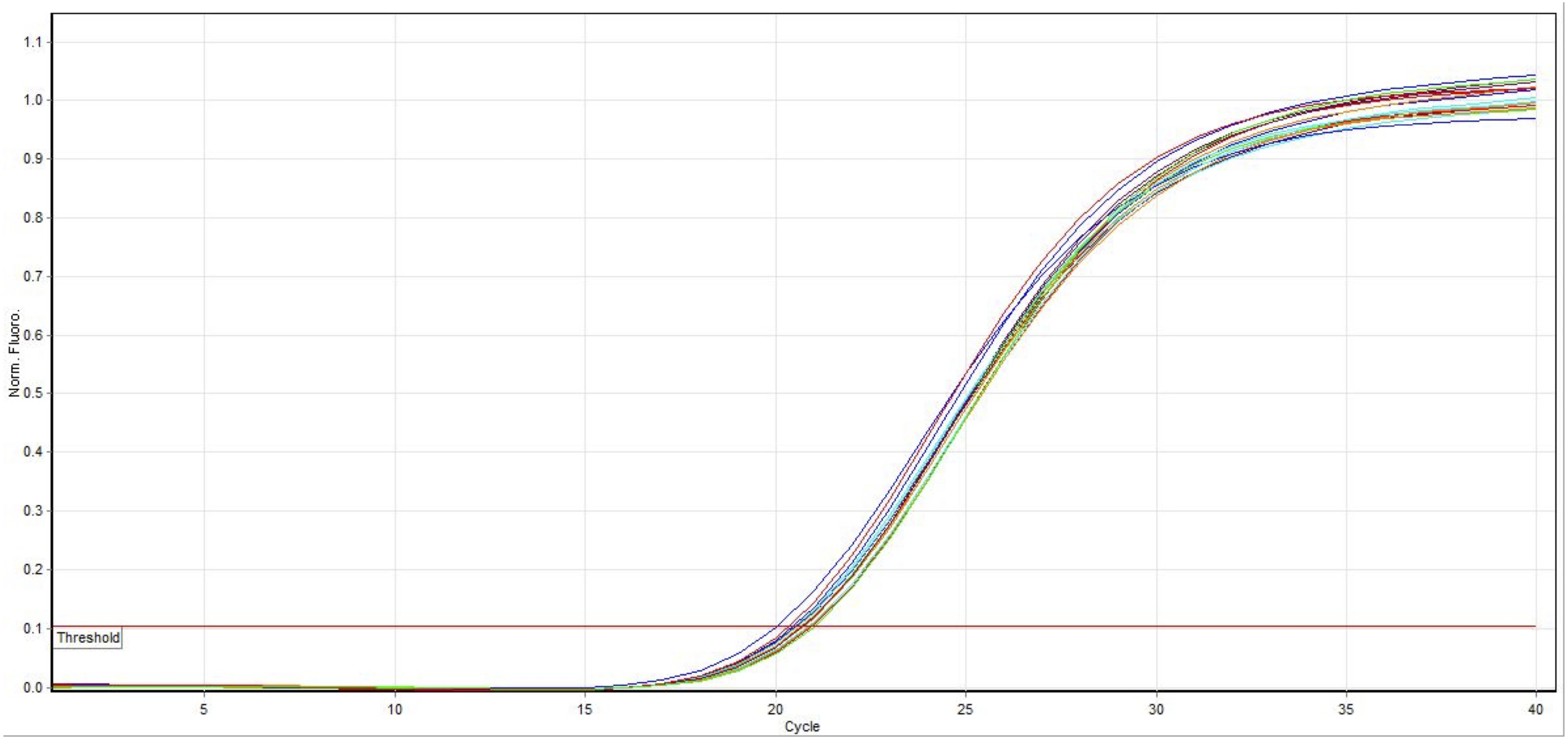

Figure S1. Amplification plot showing *DhRPS3* curves in different conditions tested. *DhRPS3* gene cycle threshold (Ct) on YPD (blue), 0.6 M NaCl (turquoise), 2.4 M NaCl (green), pH 3.5 (pink), pH 8.5 (red), and 30 mM H_2O_2 (yellow).
