# Supplementary material for: Alternative CUG Codon Usage in the Halotolerant Yeast Debaryomyces hansenii: Gene Expression Profiles Provide New Insights into Ambiguous Translation
Source: J Fungi (Basel). 2022 Sep 16;8(9):970. doi: 10.3390/jof8090970 (PMC9502446; doi:10.3390/jof8090970)
Supplement: Supplementary file 1 [file jof-08-00970-s001.zip › Figure S2.pdf]

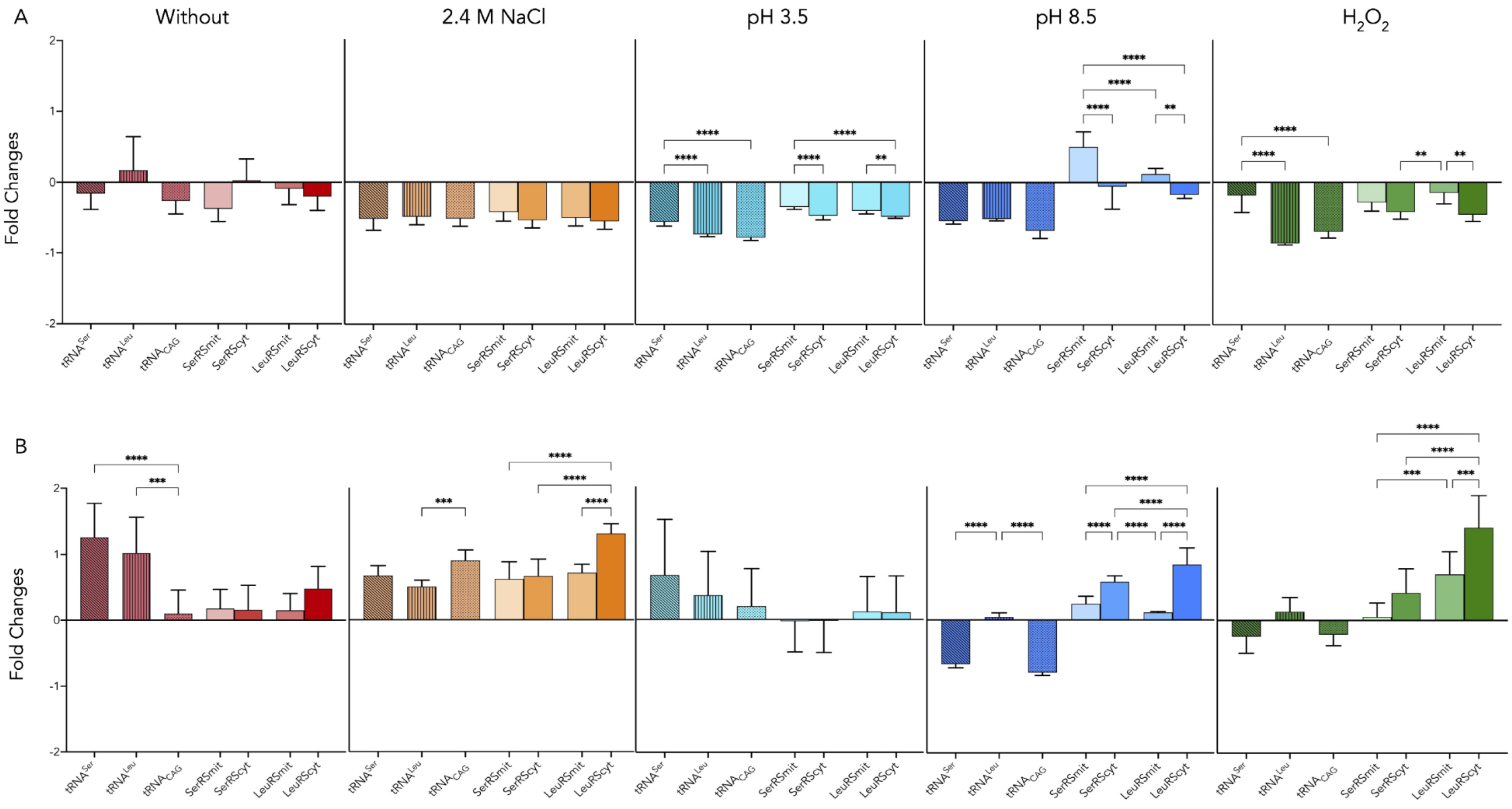

Figure S2. RT-qPCR analysis byΔΔCT method. Values normalized with *DhRPS3* gene and represented in log_2 as fold changes of $tRNA_{CAG}$, aaRS, $Trna^{Ser}$ and $tRNA^{Leu}$ genes under different stress conditions. A) Expression profile of genes compared to each other under stress conditions tested in the logarithmic growth phase. B) Expression profile of genes compared to each other under stress conditions tested in the stationary growth phase. *p*-value: 0.01 (**), 0.001 (***), 0.0001 (****).
