# Supplementary material for: Alternative CUG Codon Usage in the Halotolerant Yeast Debaryomyces hansenii: Gene Expression Profiles Provide New Insights into Ambiguous Translation
Source: J Fungi (Basel). 2022 Sep 16;8(9):970. doi: 10.3390/jof8090970 (PMC9502446; doi:10.3390/jof8090970)
Supplement: Supplementary file 1 [file jof-08-00970-s001.zip › Table S1.pdf]

Table S1. Oligonucleotides used in this work.

	Primer	Sequence (5'- 3')
P1	*LeuRScitFw*	TGCTACGAAGTCCAAGGCTG
P2	*LeuRScitRv*	CTCCAGTCAACTCTACCACCG
P3	*LeuRSmitFw*	TATGGTGGATGAACTGGCCG
P4	*LeuRSmitRv*	GCATTTTCCGCTGGTAACCC
P5	*SerRScitFw*	TGAACAAGAGCAAAAGGCCG
P6	*SerRScitRv*	ATTTGGCTTCAGCACCAGTAC
P7	*SerRSmitFw*	TCTCTGGGTCCTCGTGGTAC
P8	*SerRSmitRv*	TGTTGTTCTCCGTTTTGGTCG
P9	$tRNA_{CAG}Fw$	GATACGATGGCCGAGTGG
P10	$tRNA_{CAG}Rv$	CGACACGAGCAGGGTTCG
P11	$tRNA_{CGA}^{Ser}Fw$	GACAATGTGGCCGAGTGGT
P12	$tRNA_{CGA}^{Ser}Rv$	CGACAACAGCAGGATTCGA
P13	$tRNA_{AAG}^{Leu}Fw$	CTCTGGCCGAGTTGGTCTAA
P14	$tRNA_{AAG}^{Leu}Rv$	AGATTCGAACTCGCGCCT
P15	*DhRPS3Fw*	AAGGACCCAGCAACCAACAA
P16	*DhRPS3Rv*	AAGCGGAAGCTTCAACTGGT
