# Supplementary material for: Alternative CUG Codon Usage in the Halotolerant Yeast Debaryomyces hansenii: Gene Expression Profiles Provide New Insights into Ambiguous Translation
Source: J Fungi (Basel). 2022 Sep 16;8(9):970. doi: 10.3390/jof8090970 (PMC9502446; doi:10.3390/jof8090970)
Supplement: Supplementary file 1 [file jof-08-00970-s001.zip › Table S2.pdf]

Table S2. *D. hansenii* proteins that change their isoelectric point value by at least 0.1 from Serine to Leucine.

Protein	Gene	CTG	Size (aa)
XP_002770445.1	DEHA2E08426g	2	57
XP_457568.1	60S ribosomal protein L35	4	120
XP_459098.1	40S ribosomal protein S28	4	67
XP_457469.2	DEHA2B11858g	4	104
XP_459116.2	DEHA2D14630g	4	30
XP_457675.2	DEHA2B16632g	5	65
XP_458926.2	DEHA2D10582g	5	149
XP_458496.1	DEHA2D00638g	6	62
XP_457418.1	DEHA2B10692g	7	147
XP_459093.2	DEHA2D14146g	7	50
XP_457547.1	DEHA2B13816g	7	31
XP_457158.1	DEHA2B04510g	9	59
XP_459358.1	DEHA2E00682g	11	113
XP_456943.1	DEHA2A14102g	11	245
XP_457337.2	DEHA2B08778g	14	354
XP_459047.1	DEHA2D13178g	16	83
