# Supplementary material for: Alternative CUG Codon Usage in the Halotolerant Yeast Debaryomyces hansenii: Gene Expression Profiles Provide New Insights into Ambiguous Translation
Source: J Fungi (Basel). 2022 Sep 16;8(9):970. doi: 10.3390/jof8090970 (PMC9502446; doi:10.3390/jof8090970)
Supplement: Supplementary file 1 [file jof-08-00970-s001.zip › Table S3.pdf]

Table S3. *D. hansenii* proteins that change their isoelectric point value by at least 0.1 from Serine to Leucine tracked with AmiGO by Gene Ontology.

Protein	pI variation value	Genes	Homology	Function
XP_458926.2	0.1	DEHA2D10582g	Similar to protein P06787 from *S. cerevisiae* YBR109c CMD1	Regulates Ca^{++} independent processes (mitosis, bud growth, actin organization, endocytosis) and dependent processes (stress-activated pathways).
XP_456943.1	0.1	DEHA2A14102g	Similar to Q03406 from *S. cerevisiae* YDR489W SLD5	Subunit of the GINS complex, important in the initiation of DNA replication and is involved in the assembly of the DNA replication machinery.
XP_459047.1	0.1	DEHA2D13178g	Similar to CA0138 | CaINH1 from *C. albicans* INH1	Mitochondrial ATPase inhibitor
XP_457418.1	0.1	DEHA2B10692g	Similar to Q06213 from *S. cerevisiae* YPR168W NUT2	Subunit of the RNA polymerase II mediator complex; associates with core polymerase subunits to form the RNA polymerase II holoenzyme; transcriptional activation and basal transcription.
XP_457337.2	0.1	DEHA2B08778g	Similar to Q04651 from *S. cerevisiae* YML067C ERV41	Forms a complex with Erv46p, involved in the membrane fusion stage of transport; has homology to human ERGIC2 (PTX1) protein. Facilitates retrograde transport from the Golgi apparatus to the endoplasmic reticulum.
XP_459098.1	0.1	40S ribosomal protein S28	Similar to P02380 from *S. cerevisiae* YLR264W RPS28B	Protein component of the small (40S) ribosomal subunit.

XP_457568.1	0.1	60S ribosomal protein L35	Similar to P39741 from *S. cerevisiae* YDL191W RPL35A and P39741 from *S. cerevisiae* YDL136W RPL35B	Protein component of the large (60S) ribosomal subunit.
