# Supplementary material for: Alternative CUG Codon Usage in the Halotolerant Yeast Debaryomyces hansenii: Gene Expression Profiles Provide New Insights into Ambiguous Translation
Source: J Fungi (Basel). 2022 Sep 16;8(9):970. doi: 10.3390/jof8090970 (PMC9502446; doi:10.3390/jof8090970)
Supplement: Supplementary file 1 [file jof-08-00970-s001.zip › Table S4.pdf]

Table S4. *D. hansenii* proteins with major change in hydropathy value (Hidropathy values that varies ≥ 0.03 for Kyte & Doolittle and ≥0.3 for the two Eisenberg scales) tracked with AmiGO by Gene Ontology.

Protein	Variation value [32]	Variation value [33]	Variation value [34]	Associated gene	Homology (NCBI)	Function (AmiGO)
XP_456392.1	1	0.16	0.25	DEHA2A01232g	Similar to uniprot Q12287 *S. cerevisiae*	- Mitochondrial intermembrane space<br>- Copper chaperone activity<br>- Copper ion binding<br>- Copper ion transport
XP_457499.1	0.4	0.07	0.11	DEHA2B12518g	Highly similar to uniprot P33331 *S. cerevisiae* YER009W NTF2 Nuclear envelope protein	- Intracellular anatomical structure<br>- Transport
XP_458669.1	0.4	0.08	0.12	DEHA2D04664g	Similar to uniprot P40422 *S. cerevisiae*	- DNA-templated transcription<br>- DNA binding<br>- DNA-directed 5'-3'<br>- RNA polymerase activity
XP_459366.1	0.4	0.06	0.11	DEHA2E00858g	Similar to gnl|GLV|CAGL0K05841g *C. glabrata*	- DNA-binding transcription factor activity<br>- RNA polymerase II-specific regulation of DNA-templated transcription<br>- Nucleus<br>- Zinc ion binding
XP_457269.1	0.5	0.09	0.14	DEHA2B07150g	Highly similar to CA2973|IPF14452.repeat1 *Candida*	- Proton-transporting ATPase activity, rotational mechanism<br>- Proton-transporting ATP synthase activity, rotational mechanism<br>- Proton motive force-driven ATP synthesis<br>- Mitochondrial proton-transporting ATP synthase complex, catalytic sector F(1)
XP_459047.1	0.9	0.15	0.24	DEHA2D13178g	Similar to CA0138|CaINH1 *C. albicans* INH1	- Mitochondrion<br>- Negative regulation of ATP-dependent activity<br>- Negative regulation of nucleotide metabolic process<br>- Enzyme inhibitor activity<br>- ATPase inhibitor activity
XP_458059.2	0.3	0.05	0.07	DEHA2C08756g	Weakly similar to uniprot Q12223 *S. cerevisiae* YDL059C RAD59 Protein involved in the repair of double-	- Double-strand break repair via single-strand annealing<br>- DNA repair<br>- DNA recombination

					strand breaks in DNA during vegetative growth via recombination and single-strand annealing	
XP_458647.2	0.3	0.04	0.06	DEHA2D04136g	Similar to CA3804|CaRIM1 *C. albicans*	- Mitochondrial nucleoid<br>- Single-stranded telomeric DNA binding<br>- Single-stranded DNA binding<br>- Mitochondrion<br>- Single-stranded DNA binding
XP_456489.2	0.4	0.07	0.1	Putative mitochondrial 54S ribosomal protein RTC6	Similar to uniprot O14464 *S. cerevisiae*	No homology found
XP_457892.2	0.6	0.11	0.17	DEHA2C04730g	Weakly similar to CA2523|IPF4319 *C. albicans*	No homology found
XP_456721.1	0.3	0.05	0.08	DEHA2A08998g	Highly similar to uniprot Q02772 *S. cerevisiae* YJR034W PET191	No homology found
XP_457026.2	0.3	0.04	0.06	DEHA2B01364g	Similar to uniprot P42949 *S. cerevisiae* YJL104w PAM1	No homology found
XP_457333.2	0.3	0.04	0.07	DEHA2B08668g	Similar to CA4994|IPF2033 *C. albicans* IPF2033	No homology found
XP_458005.2	0.3	0.05	0.07	DEHA2C07436g	Some similarities with CA2824|IPF17888 *C. albicans*	No homology found
XP_458063.1	0.3	0.05	0.08	Mitochondrial 54S ribosomal protein IMG2	Weakly similar to uniprot P25642 *S. cerevisiae* YCR071C IMG2 Mitochondrial ribosomal protein of the small subunit	No homology found
XP_458072.1	0.3	0.06	0.1	DEHA2C09086g	Highly similar to uniprot Q6Q546 *S. cerevisiae* YNR032C-A HUB1 Ubiquitin-like protein modifier	No homology found

XP_458091.1	0.3	0.05	0.08	DEHA2C09438g	Similar to uniprot P53869 *S. cerevisiae* YNL211c	No homology found
XP_458277.2	0.3	0.04	0.05	DEHA2C13772g	Highly similar to uniprot O74700 *S. cerevisiae* YEL020W-A TIM9 Mitochondrial intermembrane space protein	No homology found
XP_458377.1	0.3	0.05	0.08	DEHA2C15884g	Similar to uniprot Q5KNV4 *Cryptococcus neoformans* var CNA05180 and gnl|GLV|YALI0C21789g *Yarrowia lipolytica*	No homology found
XP_458610.2	0.3	0.05	0.08	DEHA2D03278g	Weakly similar to uniprot Q08963 *S. cerevisiae* YPL213w LEA1 U2 A snRNP protein	No homology found
XP_459101.1	0.3	0.05	0.08	DEHA2D14322g	Similar to uniprot Q02771 *S. cerevisiae* YER058W PET117 Protein required for assembly of cytochrome c oxidase	No homology found
XP_459104.1	0.3	0.05	0.08	60S ribosomal protein L34	Highly similar to uniprot P40525 *S. cerevisiae* YIL052C RPL34B Protein component of the large (60S) ribosomal subunit nearly identical to Rpl34Ap and has similarity to rat L34 ribosomal protein	No homology found
XP_459358.1	0.5	0.08	0.12	DEHA2E00682g	No homology found	No homology found
XP_457675.2	0.4	0.07	0.1	DEHA2B16632g	No homology found	No homology found
XP_459093.2	0.6	0.11	0.17	DEHA2D14146g	No homology found	No homology found
XP_459116.2	0.6	0.11	0.17	DEHA2D14630g	No homology found	No homology found
XP_458415.1	0.4	0.07	0.11	DEHA2C16720g	No homology found	No homology found

XP_457158.1	0.7	0.12	0.19	DEHA2B04510g	No homology found	No homology found
XP_457278.1	0.7	0.12	0.18	DEHA2B07348g	No homology found	No homology found
XP_456695.1	0.3	0.04	0.07	DEHA2A08426g	No homology found	No homology found
XP_458496.1	0.4	0.07	0.12	DEHA2D00638g	No homology found	No homology found
XP_458602.1	0.4	0.08	0.12	DEHA2D03102g	No homology found	No homology found
XP_457547.1	1.0	0.18	0.28	DEHA2B13816g	No homology found	No homology found
XP_457581.2	0.3	0.04	0.07	DEHA2B14586g	No homology found	No homology found
XP_457070.2	0.4	0.06	0.1	DEHA2B02354g	No homology found	No homology found
XP_459460.1	0.4	0.07	0.1	DEHA2E03036g	No homology found	No homology found
XP_457089.1	0.5	0.09	0.14	DEHA2B02860g	No homology found	No homology found
XP_457615.2	0.4	0.06	0.1	DEHA2B15312g	No homology found	No homology found
XP_459330.1	0.5	0.07	0.12	DEHA2D19360g	No homology found	No homology found
XP_458622.1	0.3	0.05	0.08	DEHA2D03542g	No homology found	No homology found
XP_462639.2	0.3	0.04	0.06	DEHA2G25080g	No homology found	No homology found
